# Emergency medical dispatch services across Pan-Asian countries: a web-based survey

**DOI:** 10.1186/s12873-019-0299-1

**Published:** 2020-01-07

**Authors:** Shawn Chieh Loong Lee, Desmond Renhao Mao, Yih Yng Ng, Benjamin Sieu-Hon Leong, Jirapong Supasaovapak, Faith Joan Gaerlan, Do Ngoc Son, Boon Yang Chia, Sang Do Shin, Chih-Hao Lin, G. V. Ramana Rao, Takahiro Hara, Marcus Eng Hock Ong, For the PAROS Clinical Research Network, A. K. Sarah, M. N. Julina, G. Y. Naroo, T. Yagdir, N. Khunkhlai, P. Khruekarnchana, A. Monsomboon, T. Piyasuwankul, T. Nishiuchi, P. C. I. Ko, K. J. Song, K. D. Wong, L. P. Tham, N. Doctor, S. O. Cheah, M. Y. C. Chia, H. N. Gan, L. Tiah

**Affiliations:** 1 UNSW Medicine, University of New South Wales, Sydney, New South Wales 2033 Australia; 2 Emergency Medicine Department, National University Hospital, Singapore, Singapore; 3 Department of Acute and Emergency Care, Khoo Teck Puat Hospital, Singapore, Singapore; 4 Medical Department, Singapore Civil Defence Force, Singapore, Singapore; 5 Narenthorn EMS Center, Rajavithi Hospital, Bangkok, Thailand; 6 Southern Philippines Medical Center, Davao, Philippines; 7 Bach Mai Hospital, Hanoi, Vietnam; 8 Emergency and Trauma Department, Miri Hospital, Miri, Sarawak Malaysia; 9 Seoul National University College of Medicine, Seoul, South Korea; 10 Department of Emergency Medicine, National Cheng Kung University Hospital, College of Medicine, National Cheng Kung University, Tainan, Taiwan; 11 GVK Emergency Management and Research Institute (GVK EMRI), Secunderabad, Telangana India; 12 Graduate School of Emergency Medical System, Kokushikan University, Tokyo, Japan; 13 Department of Emergency Medicine, Singapore General Hospital, Singapore, Singapore; 14 Health Services and Systems Research, Duke-NUS Medical School, Singapore, Singapore

**Keywords:** Emergency medical services, Out-of-hospital cardiac arrest, Cardiopulmonary resuscitation, Asia-pacific, Public safety answering point

## Abstract

**Background:**

Dispatch services (DS’s) form an integral part of emergency medical service (EMS) systems. The role of a dispatcher has also evolved into a crucial link in patient care delivery, particularly in dispatcher assisted cardio-pulmonary resuscitation (DACPR) during out-of-hospital cardiac arrest (OHCA). Yet, there has been a paucity of research into the emerging area of dispatch science in Asia. This paper compares the characteristics of DS’s, and state of implementation of DACPR within the Pan-Asian Resuscitation Outcomes (PAROS) network.

**Methods:**

A cross-sectional descriptive survey addressing population characteristics, DS structures and levels of service, state of DACPR implementation (including protocols and quality improvement programs) among PAROS DS’s.

**Results:**

9 DS’s responded, representing a total of 23 dispatch centres from 9 countries that serve over 80 million people. Most PAROS DS’s operate a tiered dispatch response, have implemented medical oversight, and tend to be staffed by dispatchers with a predominantly medical background. Almost all PAROS DS’s have begun tracking key EMS indicators. 77.8% (*n* = 7) of PAROS DS’s have introduced DACPR. Of the DS’s that have rolled out DACPR, 71.4% (*n* = 5) provided instructions in over one language. All DS’s that implemented DACPR and provided feedback to dispatchers offered feedback on missed OHCA recognition. The majority of DS’s (83.3%; *n* = 5) that offered DACPR and provided feedback to dispatchers also implemented corrective feedback, while 66.7% (*n* = 4) offered positive feedback. Compression-only CPR was the standard instruction for PAROS DS’s. OHCA recognition sensitivity varied widely in PAROS DS’s, ranging from 32.6% (95% CI: 29.9–35.5%) to 79.2% (95% CI: 72.9–84.4%). Median time to first compression ranged from 120 s to 220 s.

**Conclusions:**

We found notable variations in characteristics and state of DACPR implementation between PAROS DS’s. These findings will lay the groundwork for future DS and DACPR studies in the PAROS network.

## Background

Emergency medical dispatch is an emerging area of practice and research [1]. In the beginning, dispatchers in medical dispatch services (DS’s) were typically laypeople with minimal to no training and took on a role akin to a telephone operator. The role of a dispatcher has since evolved into a crucial link in delivering patient care in emergency medical service (EMS) systems, functioning as resource allocators, non-visual clinicians and gatekeepers who are able to implement lifesaving measures prior to responders arriving on scene [1, 2], particularly in dispatcher assisted cardio-pulmonary resuscitation (DACPR) during out-of-hospital cardiac arrest (OHCA).

This evolution has occurred amidst a rapidly aging population in some countries and increased call volumes in the Asia-Pacific region, with emergency medical conditions, including OHCA, on the rise [3].

### Dispatch services in Asia and their role in out-of-hospital cardiac arrest

EMS systems in Asia are heterogenous, and remain at different phases of maturity and development [4]. Asian countries’ EMS setups are distinct from the Anglo-American and Franco-German models, being relatively underdeveloped and with a comparatively short history spanning fewer than 20 years on average [5]. Globally, DS setups and operating procedures may vary greatly, with at least 6 different models identified in Europe alone [6].

OHCA survival rates in Asia remain relatively low [7]. The most significant modifiable element correlated with better neurological outcomes post-OHCA is the time from collapse to cardiopulmonary resuscitation (CPR) and defibrillation [8, 9]. DACPR has been shown to raise survival and bystander CPR rates, and improve quality of life post-cardiac arrest [10].

This paper aims to describe the various DS’s within the Pan Asian Resuscitation Outcomes Study (PAROS) Clinical Research Network, their practices and interventions, and the state of DACPR implementation within each DS, thereby laying a foundation for future research. PAROS was set up in 2009 with the aim of improving outcomes from pre-hospital emergency care across the Asia-Pacific, and currently spans 12 countries in the region.

## Methods

We performed a cross-sectional, descriptive survey from July 2017 to March 2019. A web-based survey was disseminated to all medical directors of the DS’s within the PAROS network.

Participants were given the option to respond via either a web-based survey system or email. A designated local principal investigator at each site was responsible for verifying and accurately entering the data. The local principal investigator also responded to data queries (Additional file 1).

### Definitions

The Utstein recommendations were adopted alongside a unified taxonomy conceived by the PAROS network [11, 12]. Exclusion criteria for DACPR statistics were not finalized at the time of data collection as the revised 2017 American Heart Association quality improvement program recommendations were in the midst of being published when the survey was being disseminated [13, 14].

Key criteria for defining a DS in this study were (a) a common reporting agency or ministry, and; (b) a common operating framework and standard operating protocol, and; (c) a common service region (i.e. a city or state). Multiple dispatch centers (DCs), or physical call-centers that are responsible for taking emergency calls, may thus constitute one DS, so long as the three elements are present.

Call loads were calculated by obtaining the ratio of annual EMS transports to annual DS man hours, or the number of EMS transports activated per man hour.

### Survey tools

A standardized survey form in English was used (Additional file 2). The survey was developed by PAROS investigators. Survey domains included study site’s pre-hospital emergency care structure and characteristics, dispatcher credentials, process indicators of pre-arrival instructions and DACPR, characteristics of quality assurance program, population specific factors, and outcome measures.

## Results

**Fig. 1:**
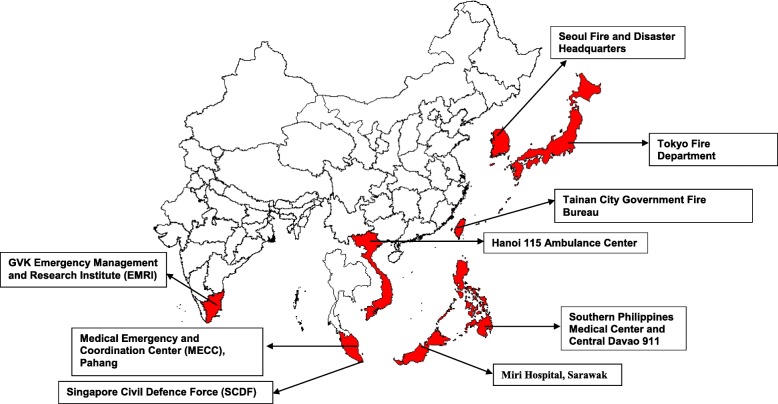
Map of Respondents’ Sites. The map was created using ArcGIS - ArcMap v10.0 (Esri, California, United States of America)

The survey was sent to 19 sites. Response rate to the survey was 47.4% (*n* = 9), with 75% (*n* = 9) of PAROS countries represented including India (1), Japan (1), Korea (1), Malaysia (1), Philippines (1), Singapore (1), Taiwan (1), Thailand (1) and Vietnam (1). These DS’s manage a total of 23 dispatch centers and serve over 80 million people in Asia (Fig. 1). 66.7% (*n* = 6) of service regions were urban. There was a large variation in call loads ranging from 0.21 to 7.66. Characteristics of each DS are listed in Table 1. Structure and staffing capabilities are listed in Table 2.

**Table 1:** Respondents’ characteristics

DS	Seoul	Hanoi	Tokyo	Miri	Davao	Tainan	Telangana	Singapore	Bangkok
Country	Korea	Vietnam	Japan	Malaysia	Philippines	Taiwan	India	Singapore	Thailand
Year Collected	2015	2016	2015	2016^a^	2016	2013^b^	2016	2016	2017
Population	9,471,871	6,452,000	13,491,000	151,500	1,632,991	1,840,257	35,003,674	5,612,253	8,306,218
Area (km^2^)	605.2	3329	2191	997.4	2444	2192	112,077	721.5	1569
Population Density/km^2^	15,651	1938	6157	152	668	840	312	7779	5294
Urbanization	Urban	Urban	Urban & Suburban	Rural	Urban	Urban	Urban, Suburban & Rural	Urban	Urban
Annual EMS Transports	335,457	23,000	1,328,054	1888	18,183	94,000	448,711	178,154	5000
Annual Total DS Man Hours	43,800	26,280	700,800	8760	87,600	35,040	233,488	219,000	17,520
Transport to Man Hour Ratio	7.66	0.86	1.90	0.22	0.21	2.68	1.92	0.81	0.29
Paramedic-Confirmed OHCA Calls	4577	1000	23,477	135	10	1119	1709	2521	207
CPR trained (%)	10	NA	7	1	10	NA	NA	30.3	NA
AED trained (%)	7.5	NA	7	1	5	NA	NA	11.1	NA
Bystander CPR (%)	52	NA	39.9	5	6	25.8	NA	54.1	34

*DS* Dispatch service, *EMS* Emergency medical service, *OHCA* Out of hospital cardiac arrest, *AED* Automated external defibrillator, *Annual Total DS Man Hours* Total number of man hours rostered annually by the Dispatch Service, *Transport to Man Hour Ratio* Number of EMS transports activated per man hour rostered by the Dispatch Service, *Paramedic-Confirmed OHCA Calls* Number of calls annually that were assessed by paramedics to be an OHCA

^a^Data was only available from April to December 2016

^b^Data was only available from 2013 due to site constraints

**Table 2:** Dispatch service capabilities & dispatcher qualifications

DS	Seoul	Hanoi	Tokyo	Miri	Davao	Tainan	Telangana	Singapore	Bangkok
Number of DCs	1	5	2	1	10	1	1	1	1
DS Configuration	Vertical	Horizontal	Horizontal	Vertical	Horizontal	Vertical	Vertical	Horizontal	Horizontal
Dispatch System	Protocol-Driven	NA	Protocol-Driven	Protocol-Driven	Guideline-Driven	Protocol-Driven	Protocol-Driven	Protocol-Driven	Guideline-Driven
Highest Level of Response	BLS + ALS	BLS	BLS + First Responder	BLS + ALS	BLS + ALS	BLS + ALS	BLS + ALS	BLS + ALS	BLS + ALS
Tiered Dispatch Response	Yes	Yes	Yes	No	No	No	Yes	Yes	Yes
First Dispatch Response	Ambulance	Ambulance	Ambulance	NA	NA	NA	Ambulance	Ambulance	Ambulance
	Fire Appliance		Fire Appliance					Fire Appliance	
			Police Car					Motorcycle	Motorcycle
			Doctor Ambulance						
DACPR	Yes	No	Yes	Yes	Yes	Yes	No	Yes	Yes
Other Pre-arrival Instructions	Yes	No	Yes	Yes	Yes	Yes	No	Yes	Yes
Medical Oversight	Yes	Yes	Yes	Yes	Yes	No	Yes	Yes	Yes
DS Standby Physician	Yes	Yes	Yes	No	Yes	No	Yes	No	No
Predominant Vocation	Paramedic	EMT	EMT	Paramedic	Nurse	Firefighter	Emergency Response Officer	Firefighter	Layperson
Minimum Entry Qualification	EMT Intermediate	BCLS	EMT Basic	EMT Basic	EMT Basic	EMT Basic	Graduate	First Aid + CPR + AED	First Aid + CPR + AED
Regularly Recertified	EMT Intermediate	BCLS	EMT Basic CPR AED First Aid	NA	EMT Basic CPR AED	NA	First Aid	BCLS AED	CPR

*DS* Dispatch service, *DC* Dispatch Centre, *Dispatch System* Nature of call interrogation, *BLS* Basic life support, *ALS* Advanced life support, *EMT* Emergency medical technician, *DACPR* Dispatcher assisted cardio-pulmonary resuscitation, *CPR* Cardio-pulmonary resuscitation, *BCLS* Basic cardiac life support, *AED* Automated external defibrillator, *Tiered Dispatch Response* Dispatch response differs based on call severity, *Other Pre-Arrival Instructions* Instructions or guidance not involving DACPR provided by dispatcher to the individuals making the emergency call, *Medical Oversight* Physician supervision of the dispatch process, *DS Standby Physician* Physician(s) physically present in the dispatch center(s) to handle difficult calls, *Minimum Entry Qualification* Minimum qualification(s) required to be deployed as a dispatcher, *Regularly Recertified* Qualification(s) required to be regularly renewed

Quality improvement indicators measured for each DS are listed in Table 3. Most survey sites (77.8%; *n* = 7) reviewed OHCA run sheets. 44.4% (*n* = 4) shared a common OHCA patient registry between the ambulance service and receiving hospitals. Almost all survey sites (88.9%; *n* = 8) tracked key EMS indicators, including the time from first contact to EMS dispatch, time from EMS dispatch to arrival at scene and time to arrival at hospital from the scene.

**Table 3:** Dispatch service quality improvement indicators

DS	Seoul	Hanoi	Tokyo	Miri	Davao	Tainan	Telangana	Singapore	Bangkok
Review OHCA Run Sheets	Yes	No	Yes	Yes	Yes	Yes	No	Yes	Yes
Sharing of OHCA Patient Data Between DS and:									
AS	Yes	No	Yes	No	No	No	Yes	Yes	Yes
RH	Yes	No	Yes	Yes	No	No	No	No	No
AS & RH in Common Registry	No	No	Yes	No	Yes	Yes	No	No	Yes
EMS KPIs Measured	Yes	No	Yes	Yes	Yes	Yes	Yes	Yes	Yes
First contact to EMS dispatch	Yes	NA	Yes	Yes	Yes	Yes	Yes	Yes	No
EMS dispatch to arrival at scene	Yes	NA	Yes	Yes	Yes	Yes	Yes	Yes	Yes
Arrival at hospital from scene	Yes	NA	Yes	Yes	Yes	Yes	Yes	Yes	Yes
OHCA KPIs Measured	Yes	No	Yes	Yes	Yes	Yes	No	Yes	Yes
EMS Time logs	Yes	NA	Yes	No	Yes	Yes	NA	Yes	Yes
DACPR Recognition Rate	Yes	NA	Yes	Yes	No	Yes	NA	Yes	Yes
Time to DACPR Recognition	Yes	NA	Yes	No	Yes	Yes	NA	Yes	No
DACPR Start Rate	Yes	NA	Yes	Yes	No	Yes	NA	Yes	No
Time to DACPR Start	Yes	NA	Yes	No	Yes	Yes	NA	Yes	No
Compression start rate	Yes	NA	No	Yes	Yes	Yes	NA	Yes	Yes
Time to first compression	Yes	NA	No	No	No	Yes	NA	Yes	Yes
Barriers to recognition	Yes	NA	Yes	Yes	No	Yes	NA	Yes	No
Barriers to compression	No	NA	No	Yes	No	Yes	NA	Yes	Yes
Patient Outcomes Measured	No	No	Yes	No	Yes	Yes	No	Yes	Yes
Pre-Hospital ROSC Rates	NA	NA	Yes	NA	Yes	Yes	NA	Yes	Yes
Hospital admission rate	NA	NA	No	NA	No	Yes	NA	Yes	Yes
Survival Rate	NA	NA	No	NA	Yes	Yes	NA	Yes	Yes
Rate of good functional recovery	NA	NA	No	NA	No	Yes	NA	Yes	Yes

*EMS* Emergency medical service, *OHCA* Out of hospital cardiac arrest, *DS* Dispatch service, *DACPR* Dispatcher assisted cardio-pulmonary resuscitation, *AS* Ambulance service, *RH* Receiving hospital, *KPI* Key performance indicator, *ROSC* Return of spontaneous circulation

77.8% (*n* = 7) of survey sites have introduced DACPR. Specific DACPR characteristics are listed in Table 4. Of the DS’s that have rolled out DACPR, 71.4% (*n* = 5) provided instructions in over one language. In 28.6% (*n* = 2) of DS’s that have introduced DACPR, not all staff were trained to deliver DACPR instructions. 85.7% (*n* = 6) of DS’s that offered DACPR provided feedback for dispatchers. All DS’s that implemented DACPR and provided feedback to dispatchers offered feedback on missed OHCA recognition. The majority of DS’s (83.3%; *n* = 5) that offered DACPR and provided feedback to dispatchers also implemented corrective feedback, while 66.7% (*n* = 4) offered positive feedback.

**Table 4:** Dispatcher assisted cardiopulmonary resuscitation characteristics

DS	Seoul	Tokyo	Miri	Davao	Tainan	Singapore	Bangkok
Country	Korea	Japan	Malaysia	Philippines	Taiwan	Singapore	Thailand
Year Introduced	2011	1994	2013	2017	2013	2011	1995
Year Data Collected	2015	2015	2016	2016	2013	2016	2017
DACPR in > 1 Language	No	Yes	Yes	Yes	Yes	Yes	No
Dispatch Staff Trained (%)	100	100	100	60	100	100	80
Script Source	Internally Developed	Internally Developed	Commercially Acquired	Internally Developed	Internally Developed	Internally Developed	Internally Developed
Computer or Card-Based	Card Based	Computer Aided	Computer Aided	Computer Aided	Card Based	Computer Aided	Card Based
DACPR Instructions	Compression Only	Compression Only	Compression Only	Compression Only	Compression Only	Compression Only	Compression Only
30:2 CPR in Specific Indications	No	Yes	Yes	Yes	Yes	Yes	No
Lookout for AEDs	Yes	Yes	Yes	Yes	Yes	Yes	No
DACPR Feedback	Yes	Yes	Yes	No	Yes	Yes	Yes
OHCA Survivors	No	No	No	NA	Yes	Yes	No
Positive Feedback	No	No	Yes	NA	Yes	Yes	Yes
Corrective Feedback	Yes	No	Yes	NA	Yes	Yes	Yes
Missed OHCA Recognition	Yes	Yes	Yes	NA	Yes	Yes	Yes

*OHCA* Out of hospital cardiac arrest, *DS* Dispatch service, *DACPR* Dispatcher assisted cardio-pulmonary resuscitation, *CPR* Cardio-pulmonary resuscitation, *AED* Automated external defibrillator

DACPR statistics for DS’s that have implemented DACPR are found in Table 5. OHCA recognition sensitivity ranged from 32.6% (95% CI: 29.9–35.5%) to 79.2% (95% CI: 72.9–84.4%). Median time to first compression ranged from 90 s to 220 s.

**Table 5:** Dispatcher assisted cardiopulmonary resuscitation statistics

DS	Seoul	Tokyo	Miri	Davao	Tainan	Singapore	Bangkok
Country	Korea	Japan	Malaysia	Philippines	Taiwan	Singapore	Thailand
Year Data Collected	2015	2015	2016^a^	2016	2013^b^	2016	2017
Paramedic-Confirmed OHCA Calls	4577	23,477	135	10	1119	2521	207
DS recognized, n (%)	2587 (56.5)	12,615 (53.7)	NA	NA	365 (32.6)	1348 (53.5)	164 (79.2)
DS recognized & compression started, n (%)	2175 (84.1)	8158 (64.7)	60	NA	43 (11.8)	1143 (84.8)	31 (18.9)
Median time to first compression (s)	174	NA	120	NA	143	220	NA

*OHCA* Out of hospital cardiac arrest, *DS* Dispatch service

^a^ Data was only available from April to December 2016

^b^ Data was only available from 2013 due to site constraints

## Discussion

Our study demonstrated many similarities and some variations in DS characteristics. Most PAROS DS’s operated tiered response systems and were protocol-driven. Medical oversight was a clear feature in most DS’s and dispatchers were predominantly healthcare providers, comprising EMTs (Emergency Medical Technicians), paramedics and nurses. DS’s were tracking quality indicators for general EMS as well as DACPR domains.

With regard to DACPR, internally developed scripts were commonplace and had been translated to the local lingua franca. All DS’s performed compression-only DACPR. OHCA recognition sensitivity and compression start rates varied considerably between DS’s.Compared to the previous survey published in 2012, Seoul, Tokyo and Singapore had transitioned from a single-tier to a tiered dispatch response system [5]. This could be a response to the aging populations they served requiring varying degrees of response [5], and overall increased call volumes. While most dispatchers had prior medical training, a protocol-driven dispatch system was predominant. In contrast to the stricter, protocol-driven dispatch systems that are algorithm-based, guideline-driven dispatch systems permit a more free-form and dynamic nature of communication [15]. Thus, the preference for protocol-driven dispatch systems may stem from concerns surrounding patient safety, as guideline-based dispatch systems require dispatchers to make more decisions, and are consequently at higher risk of poor outcomes [16].

Almost all study sites had medical oversight in place. This could be attributed to greater attention to prehospital work by stakeholders and changes in funding structures. We believe that this is beneficial for overall patient care as medical oversight has been shown to improve patient outcomes through direct influence over real-time medical decisions and formulation of dispatch guidelines and protocols [17].

Compared to 2015, measurement of quality indicators have been introduced in most DS’s and EMS systems [4]. Recent years have seen the drive towards a ‘quality-based’ culture which is encouraged by both PAROS and the Global Resuscitation Alliance [18]. Between our study’s DS’s, there remains significant differences in which performance indicators are reported, thus limiting comparisons. Continued collaborative efforts will facilitate standardization.

The increase in number of DS’s that have implemented DACPR compared to 2012 may also be attributed to Phase 2 of the PAROS study [19]. This study involved the introduction of a bundle of care to the participating PAROS dispatch services that included the implementation of a DACPR protocol and training program. Notably, in Asia, where many countries are multilingual, DACPR should be available in more than one language as language barriers are known to delay recognition of OHCA and initiation of DACPR [20], and increase dispatch times [21]. Unfortunately, this increases the staffing requirement in an already resource-limited region. In light of the potential complex multilingual environments, the preference for internally-developed scripts may be due to the need for phrasing to be simple enough to translate on-the-fly [22]. Future DACPR scripts should therefore strive to utilize simple, unambiguous and easy-to-translate language to facilitate this, in the absence of DACPR scripts in the local language.

Most DS’s surveyed provided feedback to dispatchers that was both positive and corrective. Only 2 DS’s, Tainan and Singapore, gave dispatchers information on patient outcomes. While obtaining feedback entails a greater degree of information integration with receiving hospitals, we believe this cost is well worth the effort. Dispatcher competencies in delivering DACPR are known to be partially dependent on feedback of patient outcomes [23], and having an avenue to obtain such feedback may improve rates of DACPR.

Sensitivities of OHCA recognition by PAROS DS’s that have introduced DACPR appear lower compared to Europe and American DS’s [24]. This may be due to a heterogenous population resulting in a more complex multilingual environment, further complicated by a population with relatively lower health literacy as large swathes of Asia are still developing [25]. Moreover, differences in dispatch algorithms, instructions and protocols also exist [26], and DACPR remains a fairly recent introduction within the network.

### Strengths and limitations

To our knowledge, this study is the first in-depth DS survey that sheds light on the current practices and DACPR outcomes in Asian DS’s; the cross-sectional nature of surveys is an important limitation and subject to recall bias. Different settings of DS’s (e.g. urban & rural) and the year of data reported (2013 to 2017) limit comparisons.

Some DS’s have since introduced improvements that are not captured in this survey. For example, one site only recently started DACPR, and data reported in this survey may not have reflected the improvements that have been made.

While we attempted to adopt a standardized taxonomy, our data was collected based on self-reporting and is susceptible to variability in the interpretation of questions and data points. Comparisons in DACPR statistics are also limited as the exclusion criteria were not finalized at the time of data collection.

Furthermore, although this study has attempted to compare how much call load each DS comes under by comparing the annual number of transports as a proxy for annual number of calls, to the total number of man hours rostered annually between DS’s. This does not consider the actual number of calls, as one call may have zero or multiple transports, variability in the length of calls, and how call volume varies with time of day.

There is a lack of consistent or universal metrics for assessing the call load and how it affects manpower requirements in DS’s. While existing studies on call centers frequently utilize Erlang B and Erlang C formulae to determine optimal staffing requirements, there is a paucity of research on their use in the DS setting. These remain further avenues for research.

### Future developments

Looking ahead, resource constraints may compel PAROS DS’s to capitalize on advances in technology. The growing smartphone penetration rate in Asia presents a ripe opportunity for the introduction of mobile-phone positioning systems that dispatch CPR-trained lay volunteers, such as GoodSAM and PulsePoint [27]. These efforts may increase bystander CPR rates and decrease time to first compression and defibrillation.

Additionally, this may be complemented by video-assisted dispatching, as has been trialed by GoodSAM [28]. While this implementation focused on the remote initial assessment of trauma, studies have shown that video-assisted dispatching may improve the quality of DACPR provided compared to the current, audio-instructed method [29].

The advent of artificial intelligence may also help DS’s cope with the anticipated increases in demand. For example, Singapore is looking to deploy an artificial intelligence (AI) driven speech-to-text real-time transcription solution to help reduce the time spent on collecting and transcribing information [30]. This could help reduce staffing requirements as less time may be spent per call, and potentially be used for translating information on the fly. PAROS DS’s are thus well primed to make use of these technologies to overcome their resource constraints and challenges.

## Conclusion

This is the first large-scale, network-wide assessment focusing on dispatch service characteristics and the state of implementation of DACPR within PAROS. Much regional variation between DS’s exists in terms of qualifications, QI measurements, DACPR implementation and outcome measures. These findings will lay the groundwork for future DS and DACPR studies.

## Supplementary information

**Additional file 1.** Site Principal Investigators.

**Additional file 2.** Standardized Survey Form.

## Data Availability

All data generated or analysed during this study are included in this published article and its supplementary information files.

## Abbreviations

AED: Automated External Defibrillator
ALS: Advanced Life Support
BCLS: Basic Cardiac Life Support
BLS: Basic Life Support
CPR: Cardio-pulmonary Resuscitation
DACPR: Dispatcher-Assisted Cardio-pulmonary Resuscitation
DC: Dispatch Center
DS: Dispatch Service
EMS: Emergency Medical Service
EMT: Emergency Medical Technician
KPI: Key Performance Indicator
OHCA: Out-of-hospital cardiac arrest
PAROS: Pan-Asian Resuscitation Outcomes Study
ROSC: Return of Spontaneous Circulation
