# Supplementary material for: Emergency medical dispatch services across Pan-Asian countries: a web-based survey
Source: BMC Emerg Med. 2020 Jan 7;20:1. doi: 10.1186/s12873-019-0299-1 (PMC6947813; doi:10.1186/s12873-019-0299-1)
Supplement: Supplementary file 1 — Additional file 1. Site Principal Investigators. [file 12873_2019_299_MOESM1_ESM.docx]

**Site Principal Investigators**

| DS | Name | Affiliation | Email |
| --- | --- | --- | --- |
| Bangkok | Jirapong  SUPASAOVAPAK | Narenthorn EMS Center, Rajavithi Hospital, Bangkok, Thailand | guybrush115@gmail.com |
| Davao | Faith Joan GAERLAN | Southern Philippines Medical Center, Davao, Philippines | drfjmgaerlan@gmail.com |
| Hanoi | Do Ngoc SON | Bach Mai Hospital, Hanoi, Vietnam | sonngocdo@gmail.com |
| Miri | Boon Yang CHIA | Emergency and Trauma Department, Miri Hospital, Sarawak, Malaysia | boonyang@gmail.com |
| Seoul | Sang Do SHIN | Seoul National University College of Medicine, Seoul, South Korea | sdshin@snu.ac.kr |
| Singapore | Benjamin Sieu-Hon LEONG | Emergency Medicine Department, National University of Singapore, 5 Lower Kent Ridge Road, Singapore 119074, Singapore | benjamin_sh_leong@nuhs.edu.sg |
| Tainan | Chih-Hao LIN | Department of Emergency Medicine, National Cheng Kung University Hospital, College of Medicine, National Cheng Kung University, Tainan, Taiwan | emergency.lin@gmail.com |
| Telangana | G.V. RAMANA RAO | GVK Emergency Management and Research Institute (GVK EMRI), Telangana, India | ramanarao_gv@emri.in |
| Tokyo | Takahiro HARA | Graduate School of Emergency Medical System, Kokushikan University, Tokyo, Japan | takalds@kokushikan.ac.jp |
