# Supplementary material for: Emergency medical dispatch services across Pan-Asian countries: a web-based survey
Source: BMC Emerg Med. 2020 Jan 7;20:1. doi: 10.1186/s12873-019-0299-1 (PMC6947813; doi:10.1186/s12873-019-0299-1)
Supplement: Supplementary file 2 — Additional file 2. Standardized Survey Form. [file 12873_2019_299_MOESM2_ESM.docx]

PAROS Dispatch Service Survey

Survey Description

Q1.1 Dear Respondent,
Thank you for agreeing to participate in this survey. We appreciate your response.


This survey aims to characterize emergency dispatch services present within the Pan-Asian Resuscitation Outcomes Study (PAROS) network, and how Dispatcher-Assisted Cardiopulmonary Resuscitation (DACPR) has been implemented within the dispatch service. 


For all questions which request for statistics, please provide exact values where possible, otherwise please provide your best estimate or input "NA" if not available. 


Definitions will follow the PAROS EMS Survey Definitions provided at <http://bit.ly/PAROSdefi>.

This study was reviewed and approved by the SingHealth Centralized Institutional Review Board (CIRB 2013/604/C) and National Healthcare Group Domain Specific Review Board (DSRB 2013/00929), with waiver of informed consent provided under PAROS Phase 2 (Implementation of DACPR protocol).

Demographics

Q2.1 What is the official name of your dispatch service? _____________________________

Q2.2 Which city/region does your dispatch service serve?  _____________________________

Q2.3 Which level(s) of urbanization is/are present in the service region?

☐ Urban ☐ Suburban ☐ Rural

Q2.4 How many EMS transports does your service provide annually? _____________________

Q2.5 Please complete your dispatch service's shift patterns for **weekdays** in the table below:

|  | Shift Duration (Hours) | Number of Dispatchers on Shift |
| --- | --- | --- |
| Shift 1 |  |  |
| Shift 3 |  |  |
| Shift 2 |  |  |
| Shift 4 |  |  |

Q2.6 Please complete your dispatch service's shift patterns for **weekends** in the table below:

|  | Shift Duration (Hours) | Number of Dispatchers on Shift |
| --- | --- | --- |
| Shift 1 |  |  |
| Shift 3 |  |  |
| Shift 2 |  |  |
| Shift 4 |  |  |

Q2.7 What **percentage** of the population in the service region are **CPR** trained? __________%

Q2.8 What **percentage** of the population in the service region are **AED** trained? __________%

Q2.9 What is the **annual bystander CPR** rate? __________%

Q2.10 What **year** was the aforementioned data from?  ___________

Dispatch Service Capabilities

Q3.1 How many **dispatch center(s)** are linked to your dispatch service?  ___________

Q3.2 How is the dispatch service configured?

☐ Vertical (same person attending to call and dispatching ambulance)

☐ Horizontal (one person attends to the call, another person dispatches ambulance)

Q3.3 Which **dispatch system** does your dispatch service utilize?

☐ Protocol-driven (e.g. Medical Priority Dispatch System)

☐ Guideline-driven (e.g. Criteria-Based Dispatching)

☐ No Dispatch System

Q3.4 Does your dispatch service provide any form of **DACPR** instructions?  ☐ Yes ☐ No

Q3.5 Does the dispatch center give other types of **pre-arrival instructions** (e.g. choking, trauma)? ☐ Yes ☐ No

Q3.6 What is the **highest** level of response provided by your dispatch service?

☐ Basic Life Support only

☐ Basic & Advanced Life Support

☐ Others ________________________________________________

Q3.7 Do you have a **tiered** dispatch response? ☐ Yes ☐ No

Display This Question:

If Do you have a tiered dispatch response? = Yes

Q3.8 Which of the following are part of your **first** dispatch response?

☐ Motorcycles or equivalent

☐ Fire appliances or equivalent

☐ Ambulances or equivalent

☐ Police Cars or equivalent

☐ Others ________________________________________________

Q3.9 Is there **medical oversight** of the dispatch by physician(s)? ☐ Yes ☐ No

Q3.10 Is there a standby physician **physically** in the dispatch center for difficult medical calls?

☐ Yes ☐ No

Dispatcher Qualifications

Q4.1 Which vocation is the **predominant** vocation amongst dispatchers in your dispatch center? Choose one.

☐ Firefighter

☐ Law Enforcement Officer

☐ Emergency Medical Technician (EMT)

☐ Paramedic

☐ Nurse

☐ Layperson

☐ Other ________________________________________________

Q4.2 What is the **minimum** entry qualification for dispatchers?

☐ EMT Basic

☐ EMT Intermediate

☐ EMT Paramedic

☐ Basic Cardiac Life Support

☐ CPR + AED

☐ First Aid + CPR + AED

☐ Others ________________________________________________

Q4.3 What qualification(s) are dispatchers **regularly** re-certified in? Select **all** that apply.

| ☐ CPR | ☐ AED |
| --- | --- |
| ☐ First Aid | ☐ Basic Cardiac Life Support |
| ☐ Advanced Cardiac Life Support | ☐ EMT Basic |
| ☐ EMT Intermediate | ☐ EMT Paramedic |

Dispatch Service Quality Improvement Indicators

Q5.1 Are **OHCA run sheets** reviewed?  ☐ Yes ☐ No

Q5.2 Is **OHCA patient data** shared between the dispatch service and:

|  | Yes | No |
| --- | --- | --- |
| Ambulance Service(s) | ☐ | ☐ |
| Receiving Hospital (s) | ☐ | ☐ |
| Ambulance Service(s) & Receiving Hospital(s) in a **Common Registry** | ☐ | ☐ |

Q5.3 Are **EMS performance indicators** being measured? ☐ Yes ☐ No

Display This Question:

If Are EMS performance indicators being measured? = Yes

Q5.4 Which of the following EMS performance indicators are being measured?

|  | Yes | No |
| --- | --- | --- |
| Time taken from first contact to EMS dispatch | ☐ | ☐ |
| Time taken from dispatch of EMS resource to arrival at scene | ☐ | ☐ |
| Time taken to arrive at hospital from arrival at scene | ☐ | ☐ |

Q5.5 Are **OHCA indicators** being measured? ☐ Yes ☐ No

Display This Question:

If Are OHCA indicators being measured? = Yes

Q5.6 Which of the following OHCA indicators are being measured?

|  | Yes | No |
| --- | --- | --- |
| Time logs of EMS resources (dispatch, response, turnaround times) | ☐ | ☐ |
| Does your dispatch service provide any form of DACPR instructions?  = Yes  DACPR Recognition Rate | ☐ | ☐ |
| Does your dispatch service provide any form of DACPR instructions?  = Yes  Time taken for dispatcher to recognize need for DACPR | ☐ | ☐ |
| Does your dispatch service provide any form of DACPR instructions?  = Yes  Rate of starting DACPR instructions | ☐ | ☐ |
| Does your dispatch service provide any form of DACPR instructions?  = Yes  Rate of starting chest compressions | ☐ | ☐ |
| Does your dispatch service provide any form of DACPR instructions?  = Yes  Time to first compression | ☐ | ☐ |
| Does your dispatch service provide any form of DACPR instructions?  = Yes  Barriers to recognition | ☐ | ☐ |
| Does your dispatch service provide any form of DACPR instructions?  = Yes  Barriers to compression | ☐ | ☐ |

Q5.7 Are **patient outcome indicators** being measured? ☐ Yes ☐ No

Display This Question:

If Are patient outcome indicators being measured? = Yes

Q5.8 Which of the following patient outcome indicators are being measured?

|  | Yes | No |
| --- | --- | --- |
| Return of Spontaneous Circulation in pre-hospital setting rate | ☐ | ☐ |
| Hospital admission rate | ☐ | ☐ |
| Survival rates (discharged alive or remained alive at 30 days post cardiac arrest) | ☐ | ☐ |
| Good functional recovery rate | ☐ | ☐ |

DACPR Implementation

Display This Question:

If Does your dispatch service provide any form of DACPR instructions?  = Yes

Q6.1 What year was DACPR introduced? ___________

Display This Question:

If Does your dispatch service provide any form of DACPR instructions?  = Yes

Q6.2 Do dispatchers provide DACPR instructions in **>1 language**?  ☐ Yes ☐ No

Display This Question:

If Does your dispatch service provide any form of DACPR instructions?  = Yes

Q6.3 What **percentage** of dispatch staff are trained in delivering DACPR? __________%

Display This Question:

If Does your dispatch service provide any form of DACPR instructions?  = Yes

Q6.4 Does your dispatch service utilize a **standardized script** for DACPR? ☐ Yes ☐ No

Display This Question:

If Does your dispatch service utilize a standardized script for DACPR? = Yes

Q6.5 Is the DACPR script

☐ Commercially acquired

☐ Commercially acquired and modified

☐ Internally developed

Display This Question:

If Does your dispatch service provide any form of DACPR instructions?  = Yes

Q6.6 Is the DACPR protocol for issuing instructions:

☐ Computer-aided ☐Card-based

Display This Question:

If Does your dispatch service provide any form of DACPR instructions?  = Yes

Q6.7 What is the **default instruction** in your service's DACPR protocol?

☐ Compression-only CPR ☐ 30:2 CPR

Display This Question:

If What is the default instruction in your service's DACPR protocol? = Compression-only CPR

Q6.8 Does your service's DACPR protocol include instructions for **30:2 CPR in specific indications** (e.g. asphyxial cardiac arrest)? ☐ Yes ☐ No

Display This Question:

If Does your dispatch service provide any form of DACPR instructions?  = Yes

Q6.9 Does your service's DACPR protocol include instructions asking callers to **lookout for AEDs**? ☐ Yes ☐ No

Display This Question:

If Does your dispatch service provide any form of DACPR instructions?  = Yes

Q6.10 Are dispatchers provided feedback on their DACPR performance? ☐ Yes ☐ No

Display This Question:

If Are dispatchers provided feedback on their DACPR performance? = Yes

Q6.11 What feedback is provided?

|  | Yes | No |
| --- | --- | --- |
| General positive feedback (good questioning, quick recognition, adequate guidance) | ☐ | ☐ |
| General negative feedback | ☐ | ☐ |
| OHCA survivors | ☐ | ☐ |
| Missed OHCA recognition | ☐ | ☐ |

Statistics

Q7.1 How many **EMS-confirmed, Out of Hospital Cardiac Arrest (OHCA)** calls are there in the service region annually? ___________

Display This Question:

If Does your dispatch service provide any form of DACPR instructions?  = Yes

Q7.2 Of the **paramedic-confirmed OHCA calls** (**as answered above in Q7.1**), how many were recognized by the dispatch services as **requiring DACPR**? ___________

Display This Question:

If Does your dispatch service provide any form of DACPR instructions?  = Yes

Q7.3 Of the number of paramedic-confirmed OHCA calls recognized by the dispatch service as requiring DACPR (**as answered above in Q7.2**), how many had **CPR started?** ___________

Display This Question:

If Does your dispatch service provide any form of DACPR instructions?  = Yes

Q7.4 Of the paramedic-confirmed calls recognized by the dispatch service as requiring DACPR and had CPR started (**as answered above in Q7.3**), what was the **median time to first compression** from the point of recognition?   __________s

Q7.5 Which **year** was the aforementioned data from?  ___________
